# Association of genetic polymorphisms in the interleukin-10 promoter with risk of prostate cancer in Chinese

**DOI:** 10.1186/1471-2407-10-456

**Published:** 2010-08-24

**Authors:** Jie Liu, Bao Song, Xueli Bai, Wenjian Liu, Zengjun Li, Jialin Wang, Yan Zheng, Zhehai Wang

**Affiliations:** 1 Department of Oncology, Shandong Cancer Hospital & Institute, Jinan, China; 2 Provincial Key Laboratory of radiation oncology, Shandong Cancer Hospital & Institute, Jinan, China; 3 Department of Clinical Laboratory, Shandong Provincial Hospital, Jinan, China; 4 Department of Oncology, Affiliated Hospital of Taishan Medical University, Taian, China

## Abstract

**Background:**

Recent studies identified an increased risk of prostate cancer (PCa) in Caucasian men harboring polymorphisms of genes involved in innate immunity and inflammation. This study was designed to assess whether single nucleotide polymorphisms in the IL-10 promoter play a role in predisposing individuals to PCa in a Chinese population.

**Methods:**

We genotyped three SNPs of the *IL-10 *promoter (-1082A/G, -819T/C and -592A/C) using polymerase chain reaction-restriction fragment length polymorphism analysis in 262 subjects with PCa and 270 age-matched healthy controls. Odds ratio and 95% confidence interval were determined by logistic regression for the associations between IL-10 genotypes and haplotypes with the risk of PCa and advanced PCa grade.

**Results:**

No significant differences in allele frequency or genotype distribution were observed for any of the *IL-10 *SNPs between PCa patients and control subjects. Significantly higher frequencies of -1082G, -819C and -592C allele and GCC haplotype were observed, however, in early stage patients in comparison to advanced PCa patients (for -1082 G, 13.9% vs 6.1%, OR = 2.48, *P *= 0.005; for -819 C 40.3% vs 30.8%, OR = 1.51, *P *= 0.043; for -512C, 40.3% vs 30.8%, OR = 1.51, *P *= 0.043; and for haplotype GCC 11.1%vs 5.1%, OR = 2.66, P = 0.008, respectively).

**Conclusions:**

Our results identify that *IL-10 *promoter polymorphisms might not be a risk factor for PCa in Chinese cohorts, but rather incidence of polymorphisms associates with PCa grade, suggesting that IL-10 expression may impact PCa progression.

## Background

Prostate cancer (PCa) is among the most common malignant tumors in Western males, ranking second only to lung cancer in cancer mortality [1]. PCa incidence in Asian men is significantly lower, however, the incidence of PCa in China has increased significantly in recent years [2]. While age, ethnicity, diet, and geographic factors are believed to contribute to the etiology of this disorder [3-5], genetic variations may play a role in susceptibility to PCa [6]. Recent studies suggest that genetic polymorphisms of genes involved in innate immunity and chronic inflammation, including the anti-inflammatory cytokine Interleukin-10 (IL-10), may impact susceptibility to PCa [6].

IL-10 is produced primarily by macrophages and T lymphocytes. It has important anti-inflammatory and immunosuppressive activities, including the ability to downregulate T helper 1 (Th1) cytokine and macrophage costimulatory molecule expression. The impact of IL-10 on macrophage function appears to influence blood vessel growth, as reports indicate that IL-10 may contribute to the regulation of angiogenesis in various cancers [7,8]. Due to its immunosuppressive and anti-inflammatory properties, it has been hypothesized that IL-10 contributes to tumor escape from immune surveillance, thereby enhancing tumor growth. Conversely, animal and in vitro studies demonstrate a correlation between high levels of IL-10 with smaller tumors and reduced metastasis [9].

Genetic polymorphisms and inherited factors modulate IL-10 expression. The gene encoding *IL-10 *is located on chromosome 1 (1q31-1q32), and many polymorphisms of the *IL-10 *gene promoter have been described. Examples of polymorphisms include -1082 A/G (rs 1800896), -819 T/C (rs1800871) and -592 A/C (rs1800872) in the proximal region, which influence the transcription of *IL-10 *mRNA and the expression of IL-10 in vitro [10-12]. In vitro stimulation of peripheral blood lymphocytes using concanavalin A revealed that *IL-10 *-1082 GG is associated with a 1.3-fold increase in IL-10 protein production compared to the AA genotype [13]. Similarly, the GCC haplotype exhibits significantly higher transcriptional activity than the ATA haplotype in a luciferase reporter system [14].

Multiple studies have investigated the association between IL-10 expression and incidence of PCa [15-17]. Polymorphisms of the *IL-10 *promoter at -1082 [13-15], -819 or -592 [16] reduce protein expression, and associate with an increased incidence of PCa in some reports [15-18]. In contrast, genetic variation at -592, -819, or -1082 or the promoter haplotype ATA resulted in no association in others [19,20]. These discrepancies suggest that further investigation of the association of *IL-10 *SNPs with PCa is warranted. Additionally, no such studies have analyzed the impact of these polymorphisms on PCa risk in Chinese patients. Here, we report the association of *IL-10 *polymorphisms with prostate cancer risk in a Chinese population.

## Methods

### Study subjects

The study included 262 newly diagnosed prostate cancer cases (aged 46-83 years) recruited from Shandong Cancer Hospital and Institute, Shandong Provincial Hospital and the Affiliated Hospital of Taishan Medical University between March 2006 and December 2008. The diagnosis of prostate cancer was based on digital rectal examination, serum Prostate specific antigen (PSA) concentration determination and transrectal ultrasound guided prostate biopsy. PSA concentration of 4.0 ng/mL was considered a cut-off value to carry out diagnostic work-up. Adenocarcinoma of the prostate was pathologically confirmed in all the cases, and the Gleason score (highly differentiated, score 2-5; moderately differentiated, score 6-7; poorly differentiated, score 8-10) was evaluated by pathologists working at each hospital using the Gleason scoring system [21]. The clinical T stage of the patients with PCa was evaluated according to the 2002 TNM staging system for cancer [22]. We defined more aggressive and less aggressive disease based on tumor stage and Gleason score. "Advanced prostate cancer" was defined as either a Gleason score >7 or clinical T stage >T2.

270 population controls (aged 46~81 years) were accrued from healthy volunteers who visited the three hospitals between July 2006 and July 2008 for general health exams. Controls were screened to ensure that they had never been diagnosed with cancer or other serious disease. The selected controls were matched to the cases by age (±5 years). Each subject was interviewed for family history of prostate cancer and smoking status. All subjects were unrelated ethnic Han Chinese. Written informed consent was obtained from each participant. The study was approved by the Review Boards of Shandong Cancer Hospital and Institute. Each study participant provided 2 ml peripheral blood sample.

### Genotyping

Genomic DNA was extracted from peripheral blood using Qiagen DNA Isolation Kit (Qiagen GmbH, Hilden, Germany). IL-10 promoter polymorphisms were identified by PCR amplification and restriction analysis (PCR-RFLP; Table 1). Each PCR reaction was performed in a GeneAmp PCR System 9600 thermocycler (Applied Biosystems, Foster, CA) at a final volume of 25 μl (containing 5 pmol of each primer, 50 ng genomic DNA, 1.5 mM MgCl2, 5 uM dNTPs and 1 U of Taq DNA polymerase in PCR buffer containing 10 mM Tris-HCl and 50 mM KCl). PCR cycles used were as follows: 95°C for 5 min, 35 cycles of denaturing at 95°C for 40 s, annealing at the indicated temperature for 1 min, extension at 72°C for 40 s, and a single final extension at 72°C for 10 min. The amplified products were digested with corresponding restriction endonucleases (New England Biolabs, MA, USA), and separated by electrophoresis on a 10% polyacrylamide gel stained with sliver nitrate for visualization. To confirm the genotyping results, 10% of PCR-amplified DNA samples were examined by DNA sequencing. Results between PCR and DNA sequencing analysis were 100% concordant.

**Table 1:** Primers, restriction enzymes and length of digested fragments of *IL-10 *promoter polymorphism

Polymorphism	Primer sequence	Annealing temperature	Restriction enzyme	Allele size
IL-10-1082G/A	5'-CTCGCTGCA ACCCAACTGGC-3'	58°C	*MnlⅠ*	A:139 bp
	5'-TCTTACCTATCCCTACTTCC-3'			G:106,33 bp
IL-10-819C/T	5'-TCATTCTATGTGCTGGAGATGG-3'	59°C	*Mae III*	C:125,84 bp
	5'-TGGGGGAAGTGGGTAAGAGT-3'			T:209 bp
IL-10-592C/A	5'-GTGAGCACTACCTGACTAGC-3'	58°C	*Rsa*I	C:412 bp
	5'-CCTAGGTCACAGTGACGTGG-3'			A:175,237 bp

### Statistical analysis

The SPSS statistical software package ver.13.0 (SPSS Inc., Chicago, USA) was used for statistical analysis. Demographic data between the study groups were compared by chi-square test and by Student t-test. Each polymorphism was tested for deviation from Hardy-Weinberg equilibrium by comparing the observed and expected genotype frequencies using the chi-square test. For SNP analyses, genotype and allele frequencies of *IL-10 *were compared between groups using the chi-sqare test, and odds ratios (OR) and 95% confidence intervals (CIs) were calculated using unconditional logistic regression with adjustment for age and smoking status. The linkage disequilibrium of the three loci and haplotypes of *IL-10 *(-1082,-819,-592) were conducted using the SHEsis software, from the website http://analysis.bio-x.cn/ (Bio-X Inc., Shanghai, China) [23], which uses a Full-Precise-Iteration (FPI) algorithm to reconstruct haplotypes. Additionally, the chi-sqare test was used to perform the association of IL-10 allelic and haplotype frequencies and the "advanced grade" among PCa patients. P values <0.05 were considered to be statistically significant.

## Results

### Characteristics of PCa patients and controls

A total of 262 prostate cancer cases and 270 healthy controls were recruited for the present study. All subjects were ethnic Chinese. Table 2 shows the demographic characteristics of the case and control groups. The age distributions and smoking status were similar for cases and controls. The incidence of people with a family history of PCa was higher in the case group than in the control group, however the differences were not significant (5.3% vs 2.2%, P = 0.067). The average PSA level in case patients 128.33 ± 634.21(4.0-4165.0)ng/ml was significantly higher than in controls 2.146 ± 0.954 (0.1~3.9)ng/ml (P < 0.001). Among the PCa group, 182 (69.5%) of cases had a high serum PSA level > 10 ng/ml; 161 (62.5%) cases were Gleason >7; 156 (59.5%) cases were >T2 stage. According to our grading system, there were a higher percentage of "advanced" cases (180) than "early stage" (72 cases) among the PCa patients (68.7% vs. 27.5%).

**Table 2:** Characteristics of PCa patients and controls

Characteristics	Patients, n (%) (n = 262)	Controls, n (%) (n = 270)	P
Age			
<50	8(3.0)	9(3.3)	0.729
50-59	18(6.9)	20(7.4)	
60-69	83(31.7)	86(31.9)	
70-79	123(46.9)	130(48.1)	
≥80	30(11.5)	25(9.3)	
Average age	70.7 ± 8.4	70.2 ± 8.5	
Smoking status			
Nonsmoking	102 (38.9%)	109(40.4%)	0.734
smoking	160(61.1%)	161(59.6%)	
History of prostate cancer			
Yes	14(5.3)	6(2.2)	0.067
No	248(94.7)	264(97.8)	
PSA (ng/ml)			
Average concentration	128.33 ± 634.21	2.146 ± 0.954	<0.001
≤10	80(30.5)		
>10	182(69.5)		
Gleason score			
≤7	72(27.5)		
>7	161(62.5)		
missing	29(11.0)		
Clinical T Stage			
≤T2	93(35.5)		
>T2	156(59.5)		
missing	13(5.0)		
Agressive status			
Early stage	72(27.5)		
Advanced stage	180(68.7)		
missing	10(3.8)		

### *IL-10 *gene polymorphisms and PCa

Table 3 shows the distributions of the genotypes and alleles of the *IL-10 *promoter polymorphisms. The genotype distributions for each SNP were consistent with Hardy-Weinberg equilibrium (HWE). Overall, no significant differences between PCa and control subjects were observed. Allele analyses revealed *IL-10*-1082 G in 8.4% of PCa subjects, vs. 6.1% of controls (P = 0.113). Similarly, *IL-10*-819 C and -592 C were observed in 33.6% of PCa subjects vs. 30.7% of controls (P = 0.309). Haplotype analyses were performed, and the 4 possible haplotype (ATA, ACC, GCC, GTA) frequencies are shown in Table 3. Strong Linkage disequilibrium (LD) was observed between -1082 and -819 (D' = 0.892) and -1082 and -592 (D' = 0.892). Complete LD was observed between -819 and -592 (D' = 1.0). Major ATA haplotype accounted for 68.1% and 64.9% of these four haplotypes in both of the cases and the controls, respectively. There were no significant differences in the estimated frequencies of these haplotypes between PCa patients and controls.

**Table 3:** IL-10 polymorphisms and risk of PCa

Genotype/Allele/haplotype	Controls n (%)	PCa n (%)	P	Adjusted OR* (95%CI)
Genotype(-1082A/G)				
AA	240(88.9)	222(84.7)		1.00
AG	27(10.0)	36(13.7)	0.141	1.50(0.87~2.57)
GG	3(1.1)	4(1.5)	0.520	1.68(0.35~8.17)
Allele				
A	507(93.9)	480(91.6)		1.00
G	33(6.1)	44(8.4)	0.113	1.48(0.91~2.38)
Genotype(-819 C/T)				
TT	132(48.9)	120(45.9)		1.00
TC	110(40.7)	108(41.2)	0.675	1.08(0.75~1.56)
CC	28(10.4)	34(12.9)	0.296	1.35(0.77~2.36)
Allele				
T	374(69.3)	348(66.4)		1.00
C	166(30.7)	176(33.6)	0.309	1.14(0.88~1.48)
Genotype(-592 C/A)				
AA	132(48.8)	120(45.9)		1.00
AC	110(40.8)	108(41.2)	0.675	1.08(0.75~1.56)
CC	28(10.4)	34(12.9)	0.296	1.35(0.77~2.36)
Allele				
A	374(69.3)	348(66.4)		1.00
C	166(30.7)	176(33.6)	0.309	1.14(0.88~1.48)
Haplotype				
ATA	368(68.1)	340(64.9)		1.00
ACC	139(25.7)	140(26.7)	0.551	1.13(0.86~1.47)
GTA	6(1.1)	8(1.5)	0.489	1.42(0.56~4.12)
GCC	27(5.0)	36(6.9)	0.174	1.45(0.86~2.47)

OR*, adjusted for age and smoking status

### Association of *IL-10 *polymorphisms with PCa aggressiveness

We next compared the incidence of *IL-10 *polymorphisms in prostate cancer patients in relation to their advanced status. The frequency of the G allele -1082 was significantly higher in early stage PCa patients than in advanced patients (13.9% vs. 6.1%, respectively; OR = 2.48, 95%CI 1.31~4.70, P = 0.005). At the -819 and -592 sites, the C and C alleles were also found more frequently in early stages patients than in advanced PCa patients (40.3% vs. 30.8%, OR = 1.51, 95%CI 1.01~2.26, P = 0.043; Table 4). Additionally, haplotype analysis showed that the frequency of GCC was higher in early stage patients (11.1%, OR = 2.66, P = 0.008) in comparison to advanced PCa patients (5.0%). Taken together, these data identify no significant correlation between frequency of *IL-10 *polymorphisms and incidence of PCa, however they did reveal a correlation between frequency of polymorphisms and PCa stage.

**Table 4:** Association of IL-10 polymorphisms with PCa aggressiveness

Allele/Haplotype	Early stage (72)	Advanced stage (180)	*P*	OR (95%CI)
-1082A/G				
A	124(86.1)	338(93.9)		1.00
G	20(13.9)	22(6.1)	0.005*	2.48(1.31~4.70)
-819 C/T				
T	86(59.7)	249(69.2)		1.00
C	58(40.3)	111(30.8)	0.043*	1.51(1.01~2.26)
-592 C/A				
A	86(59.7)	249(69.2)		1.00
C	58(40.3)	111(30.8)	0.043*	1.51(1.01~2.26)
Haplotype				
ATA	82(56.9)	245(68.1)		1.00
ACC	42(29.2)	93(25.8)	0.184	1.35(0.87~2.10)
GTA	4(2.8)	4(1.1)	0.128	2.99(0.73~12.2)
GCC	16(11.1)	18(5.0)	0.008*	2.66(1.30~5.45)

*P < 0.05.

## Discussion

In the present study, we analyzed the association between 3 SNPs of the *IL-10 *promoter (-1082 A/G, -819 T/C, and -592 A/C) with incidence of prostate cancer in a Chinese cohort. No significant differences in allele frequency or genotype distribution for any of the *IL-10 *SNPs were observed between patients with PCa and control subjects. However, significantly higher frequencies of the -1082G, -819C and -592C allele and GCC haplotype were observed in early stage PCa patients in comparison to advanced PCa patients.

IL-10 is a multifunctional cytokine with both immunosuppressive and anti-angiogenic functions, consequently resulting in both tumor-promoting and tumor-inhibiting properties. Multiple epidemiological studies have investigated the association between the *IL-10 *polymorphisms and the risk of different cancer types. Increased serum and peritumoral IL-10 production have been reported in many malignancies. Results with respect to PCa, however, have been inconsistent. The A allele of *IL-10*-1082 was reported by several groups to be positively associated with incidence of PCa [15-17]. Similarly, the T allele at -819 and A allele at -592 resulting in low IL-10 expression, have been associated with increased PCa risk, specifically in high grade tumors [18]. In contrast, Eder et al [19] or Michaud et al [20] report no correlation between IL-10 expression and PCa risk/status. The cause of these differences remains unclear, however several possibilities exist. Discrepancies may exist due to genetic trait differences, or the existence of distinct *IL-10 *genetic polymorphisms amongst specific populations, ethnicities and geographic regions. Furthermore, cancer is a multi-factorial disease. Individual exposures to various environmental factors in combination with genetic susceptibility may have contributed to these varied results.

We observe that the frequency of the *IL-10*-1082G allele in Chinese is similar to the frequencies observed in healthy Korean [24] and Japanese [25] populations, but significantly lower than those reported for Caucasian populations (where the frequency of *IL-10*-1082G is approximately 0.45) [18,20]. Interestingly, the frequency of -1082A (93.9%) and ATA (68%) is higher in Chinese populations than in Caucasians, yet the incidence of PCa in Chinese populations is much lower than what is observed in Caucasians. As the *IL-10 *-1082 A allele and haplotype ATA are associated with increased PCa risk in Caucasian populations, the association of these polymorphisms with PCa appears to be ethnically or geographically regulated. The -819T allele and -592A allele are in complete linkage disequilibrium in the present study. The allele ratio for -819 T/-592 A is 0.69, significantly higher than those reported for Caucasians (where the frequency of -819T and -592A are approximately 0.22 to 0.32) [18,20]. These data further confirm that *IL-10 *alleles vary significantly among ethnic groups, and warrant further investigation.

According to the International Agency for Research on Cancer (IARC), the incidence of prostate cancer in China was 1.1/10^5 ^person years (PY) in 1990 [26] and 1.6/10^5 ^PY in 2002[27]. China has the lowest incidence of prostate cancer in the world, yet the manjority of prostate cancers are not diagnosed until the advanced stages of disease progression. As serum prostate-specific antigen (PSA) screening is the only diagnostic for prostate cancer used in China diagnosis may be greatly enhanced by consideration of genetic background. Several studies have reported that the *IL-10 *-1082A allele and ATA haplotye, minor types in Caucasians but major types in China, were associated with increased PCa risk. These results, which suggest that the incidence of PCa patients in China would be greater, differ from the results of our study described here. Clearly, the association of *IL-10 *SNPs and PCa risk and progression warrant future large-scale investigation throughout multiple ethnic populations.

Here, we analyzed the association between *IL-10 *polymorphisms and PCa risks in a Chinese population. Our study revealed that there is no association between *IL-10 *SNPs and incidence of PCa, however a significantly higher frequency of -1082G, -819C and -592C alleles and GCC haplotype were observed in early stage patients compared to advanced PCa patients. To the best of our knowledge, this study is the first to assess the association of *IL-10 *gene polymorphisms with PCa for the Chinese population. Our results did not support a role for *IL-10 *SNPs in developing PCa, but as IL-10 expression alters according to cancer grade, our data suggest that IL-10 expression may impact prostate cancer progression. While the precise mechanisms by which *IL-10 *polymorphisms may modulate PCa progression remains known, evidence suggests that IL-10 modulates immune function, such as NK cell, T cells, and macrophages activity, which would alter disease progression. Additionally, increasing evidence suggests a role for IL-10 in inhibition of angiogenesis, therefore decreases IL-10 expression would de-repress angiogenic activity and promote cancer progression. Acknowledging the relatively limited sample size and for the low allelic frequencies, further studies are warranted.

## Conclusions

In conclusion, Our results suggest that *IL-10 *promoter polymorphisms might not be a risk factor of PCa development but may impact PCa progression in Chinese patients.

## Competing interests

The authors declare that they have no competing interests.

## Authors' contributions

JL and BS performed the statistical analysis and drafted the manuscript. XLB and WJL participated in the design of the study and provided clinical biospecimens. ZJL, JLW and YZ carried out the genotyping. ZHW conceived of the study, participated in its design and coordination and helped to draft the manuscript. All authors read and approved the final manuscript.

## Pre-publication history

The pre-publication history for this paper can be accessed here:

http://www.biomedcentral.com/1471-2407/10/456/prepub
